# Lectin-Based Characterization of Vascular Cell Microparticle Glycocalyx

**DOI:** 10.1371/journal.pone.0135533

**Published:** 2015-08-14

**Authors:** April K. Scruggs, Eugene A. Cioffi, Donna L. Cioffi, Judy A. C. King, Natalie N. Bauer

**Affiliations:** 1 Department of Pharmacology, College of Medicine, University of South Alabama, Mobile, Alabama, United States of America; 2 Department of Biochemistry, College of Medicine, University of South Alabama, Mobile, Alabama, United States of America; 3 Department of Pathology, West Virginia University, Morgantown, West Virginia, United States of America; 4 Center for Lung Biology, College of Medicine, University of South Alabama, Mobile, Alabama, United States of America; Brown University, UNITED STATES

## Abstract

Microparticles (MPs) are released constitutively and from activated cells. MPs play significant roles in vascular homeostasis, injury, and as biomarkers. The unique glycocalyx on the membrane of cells has frequently been exploited to identify specific cell types, however the glycocalyx of the MPs has yet to be defined. Thus, we sought to determine whether MPs, released both constitutively and during injury, from vascular cells have a glycocalyx matching those of the parental cell type to provide information on MP origin. For these studies we used rat pulmonary microvascular and artery endothelium, pulmonary smooth muscle, and aortic endothelial cells. MPs were collected from healthy or cigarette smoke injured cells and analyzed with a panel of lectins for specific glycocalyx linkages. Intriguingly, we determined that the MPs released either constitutively or stimulated by CSE injury did not express the same glycocalyx of the parent cells. Further, the glycocalyx was not unique to any of the specific cell types studied. These data suggest that MPs from both normal and healthy vascular cells do not share the parental cell glycocalyx makeup.

## Introduction

Microparticles (MPs) are submicron circulating intact vesicles that are constitutively released from a variety of cell types including endothelial cells, platelets, cancer cells, mesenchymal stem cells, and epithelial cells [1–6]. This release is increased in activated or injured cells [7–12]. The biological role of MPs is currently under intense investigation [13–18]. MPs modulate coagulation, vasoconstriction, angiogenesis, tumor metastasis, and infection [5, 12, 19–21].

Released MPs carry identifying proteins, phospholipids, and other cellular components that are indicative of the parent cell from which they are derived, making them excellent candidates for biomarkers. Frequently, identification of MPs is based on clusters of differentiation markers (i.e. CD31 for endothelial MPs) indicative of the parent cells, and expression of phosphatidylserine (PS) on their membrane [22]. While changes in the types of microparticles found in the circulation during vascular diseases such as atherosclerosis or pulmonary arterial hypertension have been reported, these studies again were dependent on clusters of differentiation or phosphatidylserine exposure [10, 23–27]. Clusters of differentiation frequently are indicative of multiple cell types, and recent work has shown that detection by PS may miss large populations of MPs that do not present PS on their outer membrane [9, 28]. Therefore, new markers of parent cell origin would be highly useful in identification of circulating MPs.

The unique carbohydrate configuration on the surface membrane of cells has frequently been exploited to identify specific vascular cell types [29–33]. Utilizing lectins, proteins known to stereospecifically target and bind sugar moieties, the glycocalyx makeup of the pulmonary artery and pulmonary microvasulature has been identified and are unique with respect to each other [34]. The glycocalyx of the aortic endothelium has been examined previously with the *Wisteria floribunda* lectin, which binds N-acetyl-D-galactosamine, however to our knowledge aortic endothelial binding to our panel of lectins has not been performed [35]. Further, *Sambucus nigra* I, has previously been used to examine pericytes, but not directly pulmonary artery smooth muscle cells, and thus to our knowledge, the glycocalyx has not been defined [31, 36]. Therefore, our goal was to determine whether cells from different regions and different vascular beds comprised unique glycocalyx signatures. With this information, we then sought to determine whether MPs released constitutively from vascular cells would mirror the unique glycocalyx properties of their parental cell type.

The glycocalyx plays a functional role in maintenance of the vascular barrier [37–39]. Injurious stimuli, such as stretch or application of neuraminidase, to the vasculature disrupt the glycocalyx and induce leak [37]. Cigarette smoke extract (CSE) induces disruption of the pulmonary endothelial cell barrier [40–42]. Thus, we also sought to determine whether CSE, as an injurious stimulus, altered the glycocalyx of the endothelium or pulmonary vascular smooth muscle and further whether any observed alterations were transferred to the resultant MPs.

## Materials and Methods

### Cell Culture

Rat pulmonary microvascular endothelial cells (MVEC), rat pulmonary artery endothelial cells (PAEC), and rat aortic endothelial cells (AOEC) were obtained from the Cell Culture Core, Center for Lung Biology at the University of South Alabama (http://www.southalabama.edu/clb/tcc/TCC.html). The isolation and characterization of these cells was performed as previously described by the core facility [34]. Rat pulmonary artery smooth muscle cells (PASMC) were obtained through a generous gift from Dr. Kurt Stenmark, University of Colorado Health Sciences Center [43]. Cells were used between passages 5–15. Endothelial cells were cultured in Dulbecco’s Modified Eagle Medium (DMEM) (10017cv, Mediatech; Manassas, VA) with 10% heat-inactivated fetal bovine serum (S11050H, Atlanta Biologicals; Lawrenceville, GA) and 1% penicillin/streptomycin solution (15140, Invitrogen; Carlsbad, CA). Smooth muscle cells were cultured in DMEM/Ham’s F-12 50/50 mixed media (10092cv, Mediatech; Manassas, VA) with 10% FBS and 0.5% gentamicin solution (1676045, MP Biomedicals; Solon, OH).

### Microparticle Collection

All cells were seeded at an initial density of 100,000/dish and grown to confluence in sterile 100 mm culture plates. Growth media was removed, and 5 mL serum-free media (SFM) was added to all plates. For cell studies with cigarette smoke extract (CSE), cells were exposed to 3% of CSE (cigarette smoke preparation described below) added directly to SFM media and incubated for 1 hour at 37°C to induce MP release. Controls were SFM only, also incubated for 1 hour at 37°C. After treatment, media was collected from the cells and centrifuged at 1000x g for 10 minutes to remove dead cells and cellular debris. The media from two plates of cells were then pooled for MP collection. The media was transferred to labeled tubes appropriate for ultracentrifugation and 2% neutral buffered formalin (53901, Thermo Scientific; Rockford, IL) was added as a fixative. The tubes were rocked at room temperature for at least 15 minutes in the fixative solution before further processing. Tubes were balanced to within ±0.02 g and ultracentrifuged (Avanti J-30i, Rotor JA-30.50 Ti, Beckman Coulter; Fullerton, CA) at 100,000 x g for 45 minutes at 4°C. Media was removed and discarded, leaving the pellet of MPs intact. MP pellets were re-suspended in buffer solution as required for further analysis.

### TEM of microparticles

A subset of microparticles were collected from pulmonary microvascular endothelial cells as described above and pipetted in culture media onto Nunc polycarbonate membranes (Electron Microscopy Sciences. Hatfield, PA) and allowed to settle onto membrane for 8 hours. Medium was then gently removed and membrane submerged in gluteraldehyde in cacodylate buffer for fixation. The specimens were then rinsed in cacodylate buffer, postfixed for 1 hour with 1% aqueous osmium tetroxide, dehydrated with graded alcohol series, and embedded in PolyBed 812 epoxy resin (Polysciences, Warrington, PA). Thick (1 μm) sections were cut with glass knives and stained with 1% toluidine blue and examined via light microscopy for “structure orientation.” Thin (80 nm) sections cut from the same block with diamond knives and examined and photographed with a Philips CM 100 transmission electron microscope (FEI, Hillsboro, OR).

### Cell Collection

Once microparticle-rich media was removed from cells, 1 mL trypsin was added to each plate and plates were incubated at 37°C for 5 minutes. Then, 1 mL cell culture media containing FBS was added to neutralize the trypsin. Cells were removed from the plate, added to sterile 2mL microcentrifuge tubes and centrifuged at 1000 x g for 10 minutes. The supernatant was removed and the cells were resuspended in 1.5 mL PBS containing 2% neutral buffered formalin. The cells were rotated for at least 15 minutes in the fixative solution before further processing. The cells were again centrifuged at 1000 x g for 10 minutes, the supernatant was removed, and the cells were re-suspended in 1 mL buffer solution as required for further analysis.

### Cigarette Smoke Extract Preparation

The apparatus was assembled using tubing and a 250mL sidearm vacuum flask in a ventilated hood. The smoke from 20 research cigarettes (3R4F, University of Kentucky Tobacco and Health Research Institute; Lexington, KY), with filter removed, was bubbled through 200 mL sterile PBS. Each cigarette was burned for five minutes under constant negative pressure. The resulting pH of the CSE solution was adjusted to 7.4 using sodium bicarbonate (S5761, Sigma; St. Louis, MO) and passed through a 0.22 μm filter (SCGPT05RE, Millipore; Billerica, MA) to remove large particles. In order to determine the consistency of CSE preparation, absorbance of the extract was taken at 230nm. The absorbance measured between 3.18 and 3.28 was considered 100% concentration and diluted accordingly for cell treatment. Aliquots of the extract were added to microcentrifuge tubes to be stored at -20°C until needed. When adding CSE to cell culture plates, an aliquot was thawed at room temperature and added to pre-warmed cell culture media. The FLICA Caspase 3/7 assay kit (ImmunoChemistry Techonologies, Bloomington, MN) was used to determine caspase activity in CSE treated cells (S2 Fig). Staurosporine was used as a positive control (1μM for1 hour, Calbiochem). The assay was performed using the manufacturer’s instructions.

### Addition of Lectins to MPs and Cells

Previously collected MPs were resuspended in 500 μL sterile, 0.1 μm-filtered PBS. Cells were resuspended in 1 mL sterile PBS. The lectin of interest was added directly to the suspended EMPs and cells in a darkened room. Lectin dilution used for MPs was 1:100 and for cells was 1:200. The FITC-labeled lectins studied include: *Sambucus nigra* (SNA1), *Maackia amurensis* (MAA), *Griffonia simplicifolia I* (GS1), and *Helix pomatia* (HP). Then, the tubes were wrapped in foil and gently rocked for 20 minutes. To rinse MPs of extraneous lectin, 10 mL of filtered PBS was added to the tubes and they were centrifuged at 100,000xG for 45 minutes. The MP pellets were then resuspended in 1 mL PBS and transferred to labeled, sterile FACS tubes (BD Falcon Polystyrene 5mL, BD Biosciences; Bedford, MA) to be analyzed by flow cytometry. Cells were washed by adding 800 μL sterile filtered PBS to tubes containing cells and lectin solution. Cells were spun at 1000 x g for 10 minutes, supernatant removed, then washed once more. After the final wash buffer was removed, cells were re-suspended in 1 mL sterile PBS and transferred to labeled FACS tubes for further analysis by flow cytometry.

### Flow Cytometry

Microparticles and parent cells were analyzed by flow cytometry (BD FACSCanto II, BD FACSDiva Software). For microparticle analysis, logarithmic amplification of forward light scatter (FSC-Area), sideward light scatter (SSC-A), and fluorescent light scatter (FITC-A) were used to identify populations. Threshold triggers were based on both FSC-A and SSC-A to minimize instrument noise and were set at the lowest possible setting for the instrument (200). Before each experiment, the flow cytometer was calibrated with 1 μm Fluoresbrite plain microsphere calibration beads in PBS (21636, Polysciences Incoporated; Warrington, PA). Forward-scatter voltage was adjusted so the 1 μm peak stayed within a constant FSC-A range. Analysis is limited to particles 1 μm and under. Particle counts were obtained for each sample and normalized with the addition of a known amount of CountBright fluorescent counting beads (7 μm diameter, Molecular Probes; Eugene, OR) per sample volume. The counting beads appear in the upper right region of a standard plot and are gated. We collected 2000 bead events per sample to assure a statistically significant determination of sample volume. The total number of MPs that were fluorophore-positive was calculated based on the unstained control. This positivity indicates covalent bonding of the lectin to the glycocalyx of the MPs, which in turn indicates that the specific carbohydrate linkage is present. The ratio of lectin positivity to EMP counts was determined for CSE-treated, an untreated control, and an unstained control. Cytometric analysis was performed using forward scatter vs. side scatter plots and histograms (S1 Fig).

### Statistical analysis

Statistical significance was determined by ordinary One-Way ANOVA with Tukey’s multiple comparison test. The confidence interval for all samples was 95%. All data reported are at least n = 3 independent experiments and error bars represent SEM.

## Results

### Microparticle imaging

**Fig 1:**
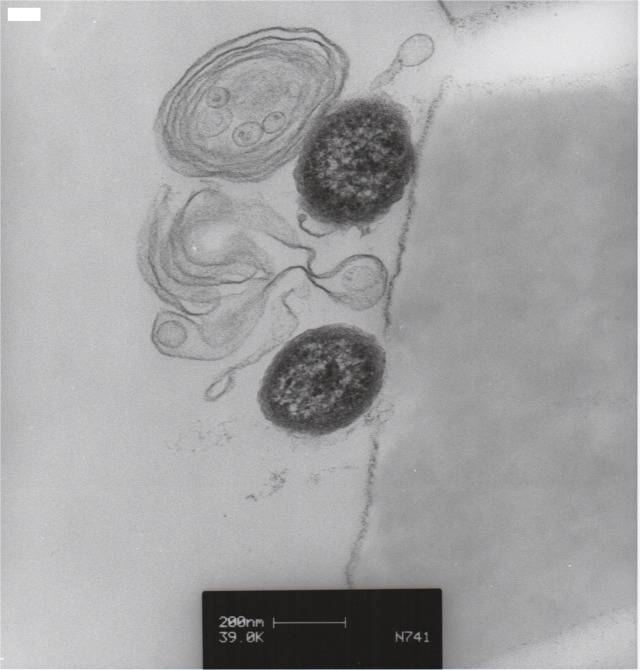
TEM of Microparticles. MVECs were seeded and grown to confluence on 100mm dishes. At confluence media was collected and centrifuged for MP collection and placement on filters as described in methods. TEM images of MPs were taken. These images illustrate that our collection protocol isolates MPs of the appropriate size.

In order to confirm that our protocol for MP isolation resulted in retrieval of intact submicron vesicles we performed transmission electron microscopy. As a representative cell type to illustrate our MP collection protocol we used MVECs. MVECs were grown to confluence and cell culture media was collected and centrifuged as described in methods. Fixation and TEM were performed as described previously [44]. Using this method we clearly show that our preparation isolates MPs of the appropriate size range and as intact vesicles. While the intravesicular structures of MPs remain unknown, it is evident that the vesicles contain membrane and likely cytoskeletal features (Fig 1).

### The glycocalyx profile of vascular cells

In an effort to characterize the glycoprofile of the vascular cells in this study we used a panel of 4 lectins: *Griffonia simplicifolia* (GS1) *I*, *Helix pomatia* (HP), *Sambucus nigra* (SNA1), and *Maackia amurensis* (MAA). Each of these has binding specificity for terminal chain carbohydrates on the surface of the cells (Table 1). Since there is significant heterogeneity in both expression of surface molecules and the physiologic function of endothelium of pulmonary and systemic vasculature, we examined cells from both. The vascular cells we examined were pulmonary microvascular endothelial cells (MVECs), pulmonary artery endothelial cells (PAECs), aortic endothelial cells (AOECs), and pulmonary artery smooth muscle cells (PASMCs) as a non-endothelial vascular cell type.

**Table 1:** Lectin panel used for all studies.

*Lectin*	*Full Name*	*Specificity*	*Order Information*
MAA	Maackia amurensis lectin	α-2,3 sialic acid	F-7801-2 –EY Labs^1^
SNA I	Sambucus nigra (Elderberry) lectin	α-2,6 sialic acid	F-6802-1^1^
GS1	Griffonia simplicifolia I	α-D-galactosyl moieties	F-2401-2^1^
HP	Helix pomatia lectin	α-D-sialic acid	F-3601-1^1^

^1^ EY Laboratories; San Mateo, CA.

**Fig 2:**
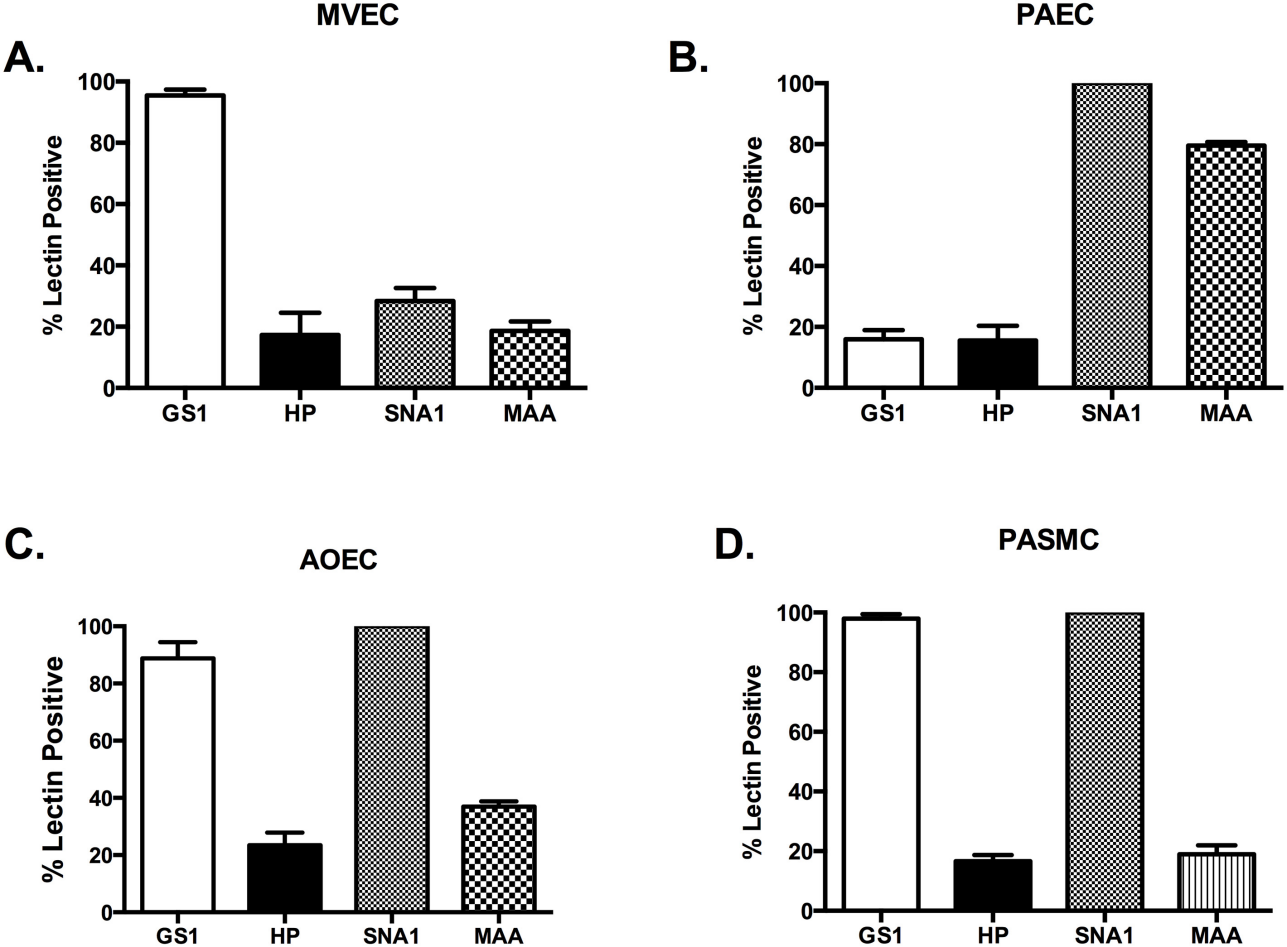
Vascular cells from the macrocirculation have specific SNA lectin binding. All cell types were grown to confluence, media changed to serum free media for 1 hour, trypsinized, and stained for our lectin panel in Table 1 as described in methods. (A) MVECs show preferential binding for *Griffonia simplicifolia* (GS1) lectin as previously reported (95.43 ± 1.9%). (B) PASMCs have significantly more binding to *Sambucus nigra* (SNA1) and *Maackia amurensis* (MAA) than GS or *Helix pomatia* (HP) (100 and 79.43 ± 1.2% vs. 15.95 ± 3.0 and 15.58 ± 4.8%, respectively; P<0.05). (C) AOECs preferentially bind GS1 and SNA1 compared to HP and MAA (88.7 ± 5.7 and 100% vs. 23.48 ± 4.4 and 36.9 ± 1.8%, respectively. *P<0.05). (D) PASMC bind GS1 and SNA1 preferentially (97.9 ± 1.5 and 100% vs. 16.6 ± 2 and 18.93 ± 3.0%, respectively. P<0.05).

While the microvasculature of the pulmonary circulation in culture and in isolated lung examinations is GS1 positive and our results are the same, we also used HP, SNA1, and MAA on our cells [34, 45]. We found that less than 20% of the cells stained positive for any other member of the lectin panel (Fig 2A). Alternatively, the PAECs stained 100% positive for SNA and nearly 80% positive for MAA, while staining less than 20% positive for GS1 or HP (Fig 2B). These data would suggest that the MVEC and PAEC express unique and identifiable glycocalyx linkages.

We were unable to find previous reports of specific AOEC lectin profiling. We found that AOEC stained 100% positive with SNA1 and greater than 80% stained positive for GS1, indicating significant expression of α-D-galactosyl moieties and α-2,6 sialic acid. However, only 20% of cells were HP positive and 30% were MAA positive (Fig 2C). Therefore, it seems that AOEC have glycocalyx similarities to both the pulmonary microvascular and macrovascular cell types. Interestingly the PASMC stained nearly identical to the AOEC. The PASMCs were 100% positive for SNA and nearly 100% for GS1 (Fig 2D).

### Glycocalyx of constitutively produced MPs from vascular cells

**Fig 3:**
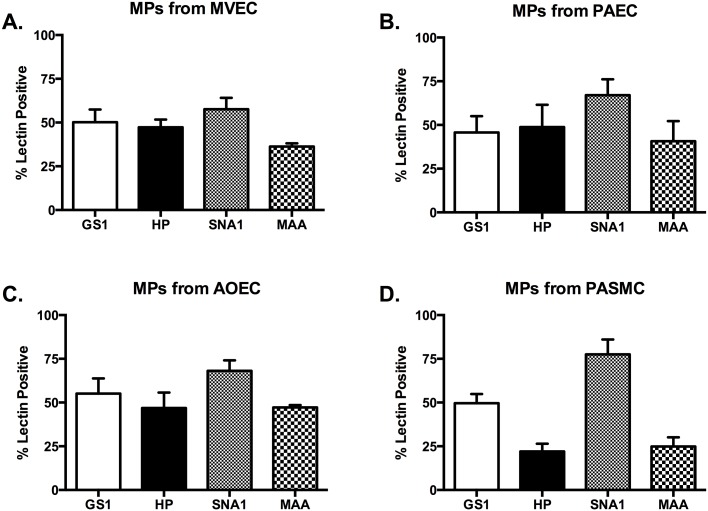
MPs released constitutively from vascular cells do not recapitulate the glycocalyx of the parent cells. All cell types were grown to confluence, media changed to serum free media for 1 hour. Media was then centrifuged as described to collect MPs. MPs were then stained with the lectin panel in Table 1. The MPs released from MVECs, PAECs, and AOECs (A, B, and C) do not show any significant positivity for any of the lectins studied (all ranging from 40–60% positive. P = not significant). The PASMC-MPs were significantly positive for SNA over MVEC-MPs and PAEC-MPs, but not AOEC-MPs (78.53 ± 4.5 vs. 57.66 ± 6.6 and 67.1 ± 9.06, respectively. P<0.05).

In order to determine whether the glycocalyx of MPs would be representative of their parent cells, we collected MPs from cells in growth media. We analyzed MPs via flow cytometry with the same panel of lectins as the parent cells. Our first analysis was of MPs from MVECs. We found that while the MPs were more than 50% positive for GS1 and SNA1, they were also positive for HP and MAA at 47% and 36%, respectively (Fig 3A). None of the positive lectin staininig on MVEC-MPs was statistically unique, however it is intriguing that the glycocalyx differs from the parent cells.

Next we analyzed MPs from the PAECs. While most were all above 40% positive, only SNA1 stained 67% positive (Fig 3B). This was not however statistically significant among the lectins analyzed suggesting that no one lectin might be used to identify PAEC specific MPs. Further, when compared to MPs from MVECs there is no statistical significance. Lastly, similar to the MVEC-MPs, the staining does not match that of the parental cell.

Our analysis of MPs from AOECs revealed that they were strongly positive with GS1 and SNA1, significantly more so than HP and MAA (Fig 3C). Of the cell types analyzed this microparticle profile most closely resembles that of the parent cells, with AOECs being 88% and 100% positive for GS and SNA1, respectively. However, while within the AOEC-MP population statistical significance was achieved, compared to MPs from either MVECs or PAECs there was not significance.

MPs from PASMCs were analyzed as well and found to be significantly positive for SNA1, they were also positive for GS1 (Fig 3D). For both HP and MAA staining the MPs from PASMCs were less than 30% positive (Fig 3D). While this appears to correlate with the lectin membrane staining of the PASMC parent cells, and the SNA1 staining of PASMC-MPs is statistically significant compared to MPs from either MVECs or PAEC, the SNA1 staining does not discriminate PASMC-MPs from AOEC-MPs.

**Fig 4:**
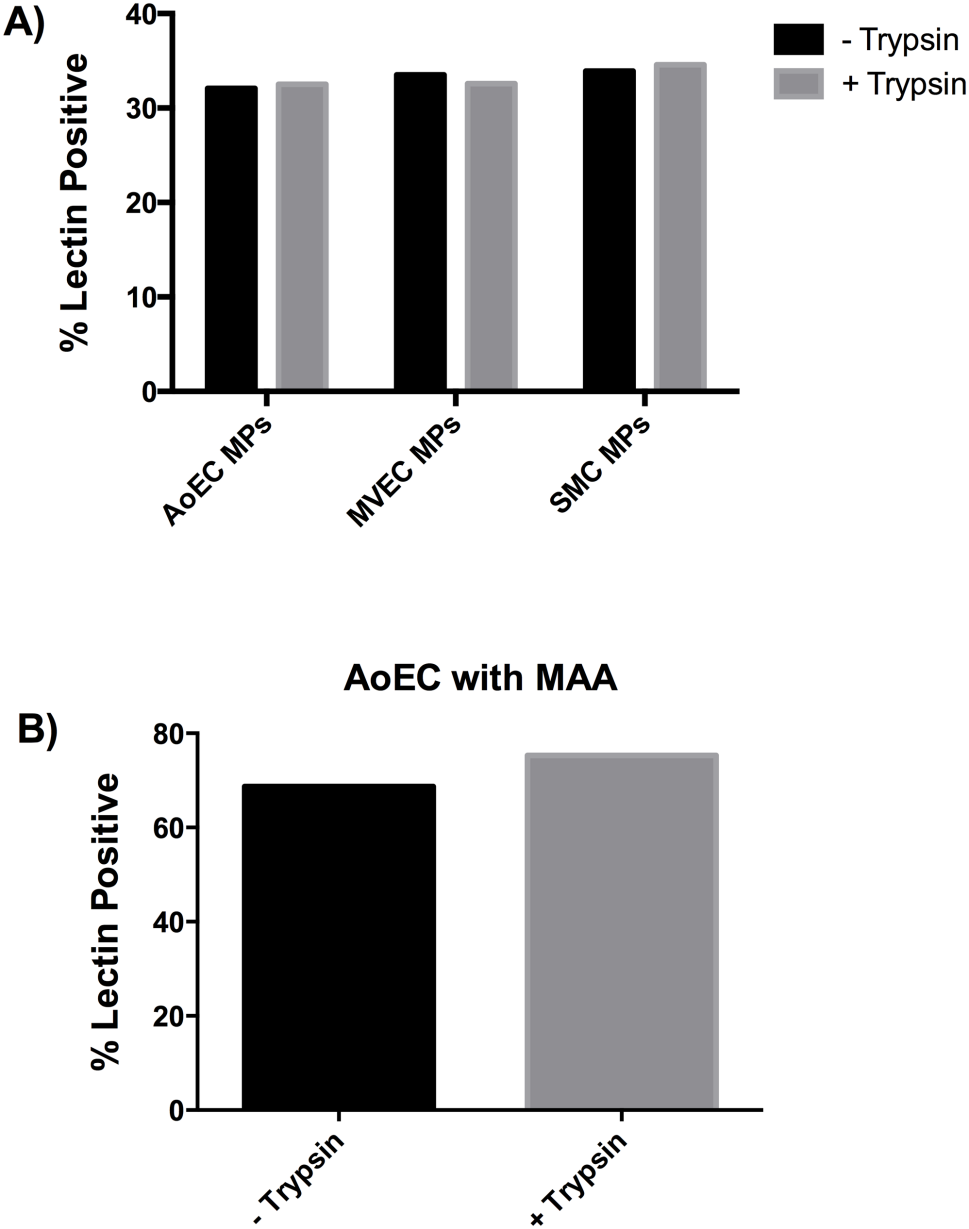
Lectin staining not affected by trypsin treatment. MPs were collected from serum-free media of confluent cells as described in methods. Prior to formalin fixation MPs were re-suspended in trypsin for 5 minutes. A) Microparticles isolated from media of AOECs, MVECs, and PASMCs were untreated or treated with trypsin and stained with MAA. In Figs 2 and 4 MAA labels approximately 30% of the MPs and this was not altered by trypsin treatment. B) AOECs were manually dissociated using a cell scraper or exposed to trypsin followed by staining with MAA. There is no significant difference in staining between the two dissociation methods.

Since our protocol for the lectin characterization of all cell types indicates the use of trypsin to remove cells from the culture dish, and trypsin may alter the glycocalyx, we also treated MPs from AOECs, MVECs, and PASMCs with trypsin. We analyzed the MPs with MAA and found that there was no change in MAA sensitivity either before of after trypsin treatment (Fig 4A). We also used manual disruption of the AOECs and compared MAA sensitivity to trypsin treatment. There was no change in MAA staining (Fig 4B).

### Inhibition of lectin binding

**Fig 5:**
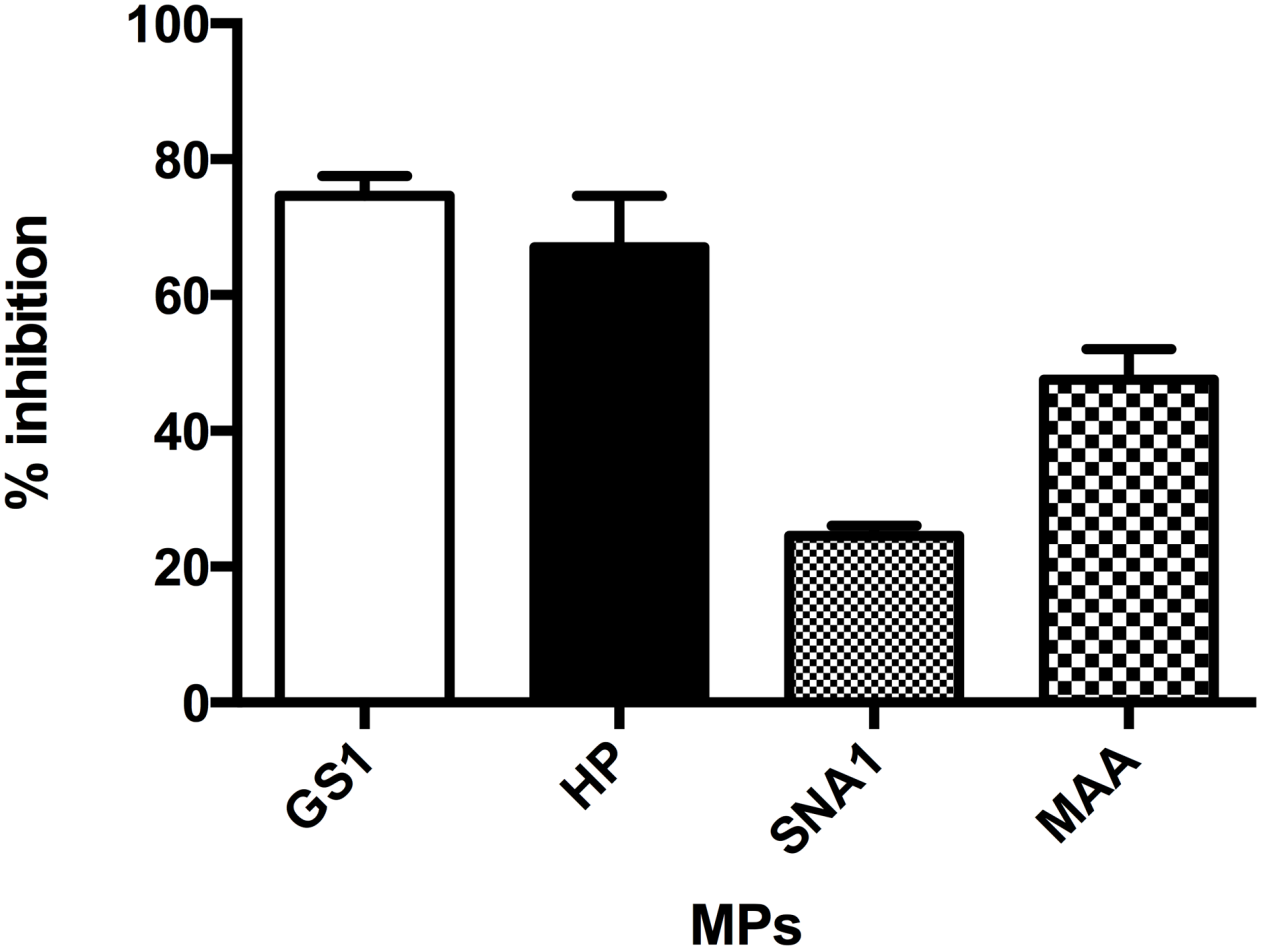
Lectin staining is inhibited by treatment with sugar. The lectins HP, SNA, MAA, and GS1 were presaturated with the sugar D(+)galactose. MPs isolated from MVECs were then stained with lectins or Dgal saturated lectins. Dgal significantly inhibited all lectin binding to the MPs. (HP 37 ± 5 vs. 13 ± 4; SNA 68 ± 2 vs. 51 ± 0.3; MAA 34 ± 0.6 vs. 18 ± 1; and GS1 54 ± 6 vs. 13 ±1; stained vs. Dgal saturated respectively, n = 3–5 per group P< 0.001).

To determine lectin specificity we used MPs isolated from unstimulated MVECs. We chose D(+)galactose as the inhibitory sugar based on recommendations from the manufacturer and our protocol is based on previous work in the lectin field [46, 47]. Pre-saturated lectins were then used to stain MPs isolated from MVECs. We found that D(+)galactose treatment did significantly inhibit HP, SNA, MAA, and GS1 binding suggesting specificity (Fig 5).

### Glycocalxy of injured vascular cells

Cigarette smoke extract induces endothelial dysfunction and increased permeability in pulmonary endothelial monolayers [42, 48, 49]. Thus, cigarette smoke is considered an injury stimulus for the vasculature. Further, exposure to cigarette smoke increase circulating endothelial MPs in human studies [50]. In our studies, as a representative cell type we chose the MVECs and examined caspase-3 activity as a marker of apoptosis in the presence of cigarette smoke extract. Treatment of MVECs with cigarette smoke extract stimulated increased caspase-3 activity compared to untreated cells (S2 Fig). We then used cigarette smoke treatment of our cultured vascular cells to determine whether this type of injury altered the glycocalyx of the parent cells.

**Fig 6:**
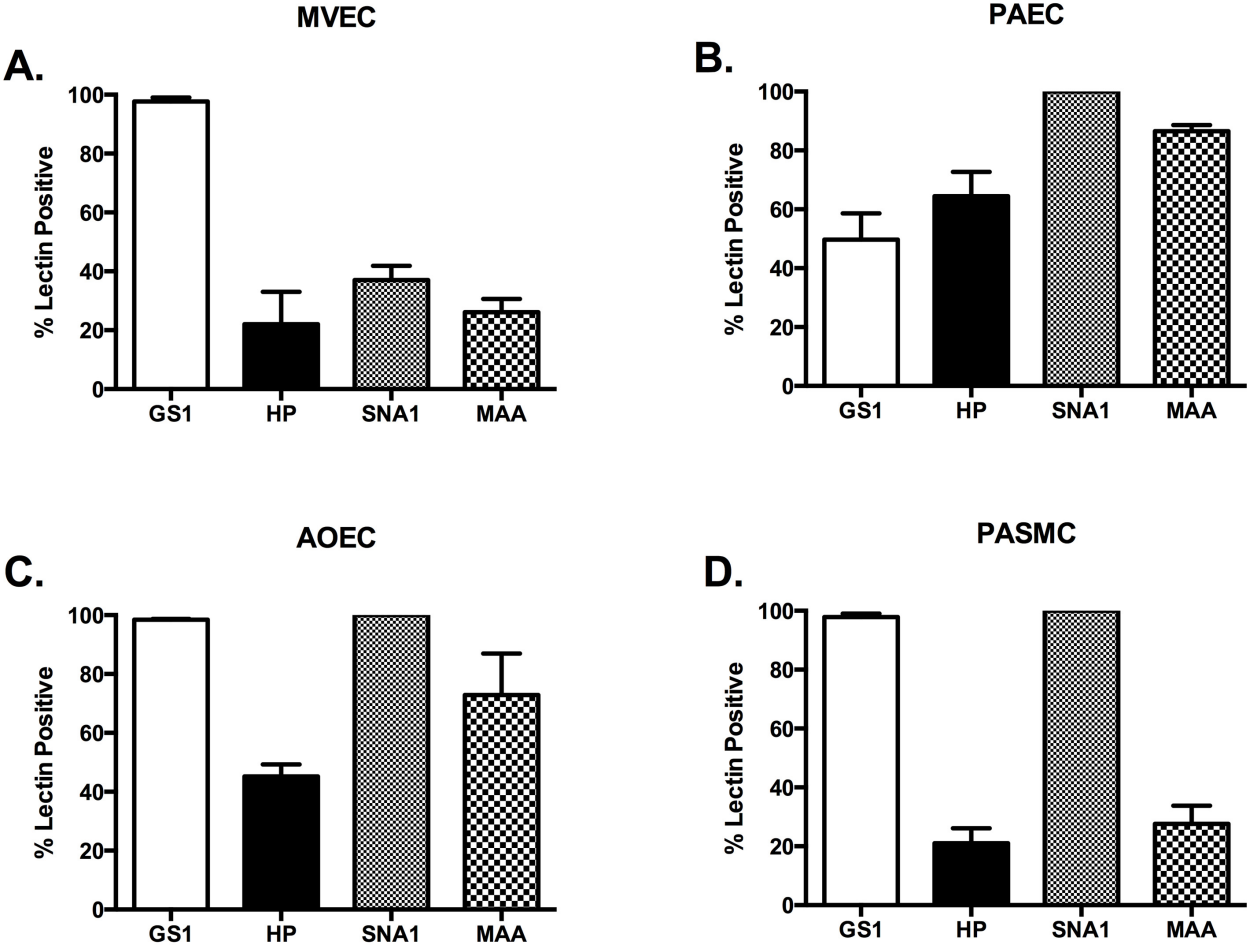
Glycocalyx of cigarette smoke extract injured vascular cells. All cell types were grown to confluence, media changed to serum free media for 1 hour in the presence of 3% CSE, trypsinized, and stained for our lectin panel in Table 1 as described in methods. (A) MVECs treated with 3% CSE have no significant changes in their glycocalyx profile compared to healthy cells. (B) Injury of PAECs with 3% CSE induces increased lectin binding of GS1 and HP (15.95 ±3.0 vs. 49.67 ±8.92 for GS and 15.58 ± 4.8 vs. 64.47 ± 8.2 for HP), but there was no significant change to SNA or MAA. (C) AOECs treated with 3% CSE had no change in GS1 or SNA1 binding, however both HP and MAA increased significantly (23.48 ± 4.4 vs. 45.20 ± 4.1 for HP and 36.9 ± 1.8 vs. 72.83 ± 14.09 for MAA. P<0.05). (D) There were no significant differences in lectin binding in CSE treated PASMCs.

Using a concentration of 3% cigarette smoke extract (CSE) monolayers were treated for one hour. With the MVECs we found that there were no significant changes in staining when compared to untreated cells. GS1 staining remained above 95% positive and all others at or below 30% positive (Figs 2A and 6A; n = 3). We next examined the PAECs treated in the same manner. Interestingly, PAECs responded to CSE treatment with a significant increase in both GS1 and HP staining, 50% and 65% respectively (Figs 2B and 6B; n = 3). However, there was no change in the SNA1 and MAA positivity between untreated and CSE treated PAECs. The AOECs treated with CSE were significantly more positive for HP and MAA compared to control, however GS1 and SNA1 stayed the same (Figs 2C and 6C; n = 3). Lastly, the PASMC had no significant changes in their lectin profile when compared to untreated cells (Figs 2D and 6D; n = 3).

### Glycocalxy of MPs from injured vascular cells

While cigarette smoke is known to induce vascular injury and induce the release of MPs, currently nothing is known about the effects of injury on the MP glycocalyx. We used our lectin panel to identify whether cellular injury altered the glycocalyx of the MPs released in the presence of CSE.

**Fig 7:**
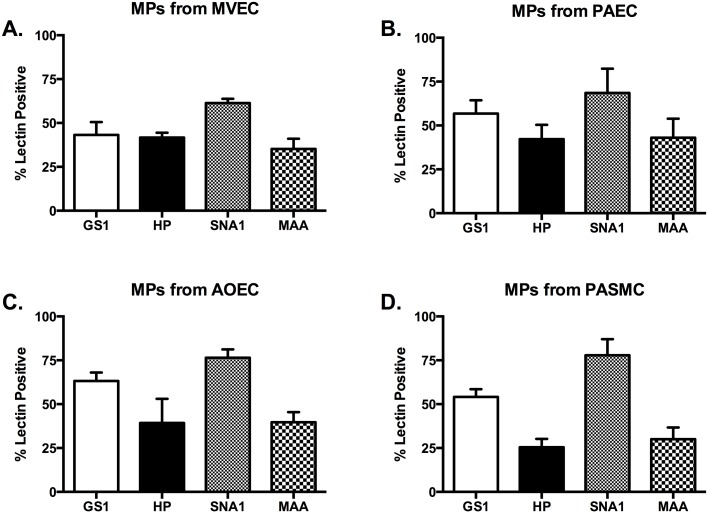
Glycocalyx of MPs from cigarette smoke extract injured vascular cells. All cell types were grown to confluence, media changed to serum free media for 1 hour in the presence of 3% CSE. Media was then centrifuged as described to collect MPs. MPs were then stained with the lectin panel in Table 1. The lectin staining of MPs isolated from MVECs, PAECs, AOECs, and PASMCs (A, B, C, and D) treated with 3% CSE were not significantly different from MPs isolated from the control cells. Thus, CSE injury to the parent cell does not seem to significantly alter the glycocalyx of the released MPs.

MPs from MVECs were collected and analyzed following cell treatment with 3% CSE. Using our lectin panel we did not find any differences in the glycocalyx between MVEC-MPs from control or CSE treated cells. However, the SNA1 staining of CSE MPs was significantly higher than the MAA staining (Figs 3A and 7A; n = 3–8 per group). We next examined MPs from PAECS and there were no significant differences among the different lectins, nor was there any significant difference when compared to MPs from untreated PAECs (Figs 3B and 7B; n = 3–5 per group). MPs from CSE treated AOECs were similar to control in that SNA staining was significantly higher than MAA, however in MPs from untreated cells there was also significant differences between SNA1 and HP (Figs 3C and 7C; n = 3–9 per group). Therefore, the MPs from AOECs did change slightly with CSE treatment of the parent cells. Lastly, MPs from PASMCs treated with CSE had a similar pattern to MPs from untreated cells. SNA1 staining remained above 75% and was significantly higher than both HP and MAA (Figs 3D and 7D; n = 3–5 per group). Although GS1 staining increased in the MPs from injured PASMC, this difference was not significant from control.

## Discussion

Current methods of vascular cell MP detection are focused on expression of clusters of differentiation markers to identify the parent cell. However, these CD molecules are frequently expressed on multiple cell types and provide no information on the vascular segment from which the MPs may be derived. While MPs are gaining recognition as indicators of vascular injury, information on the specific segment of injury would be beneficial for targeting therapies. Therefore, we sought to determine whether the use of lectins might better discriminate vascular segment derivation of MPs.

The panel of lectins chosen was based on previous data that the GS1 and HP, in particular may have the ability to discriminate between micro- and macrocirculation, respectively [34]. We expanded this profile to include SNA and MAA. The cells used were representative of the pulmonary and systemic circulations and incorporated both large and small vessels. In our initial analysis of the cell types alone we found that our MVEC data correlated with previous reports of nearly 100% positive for GS1, and significantly less HP, SNA1 or MAA. However, our PAEC data differ from previous reports of significant staining for HP [34]. While the reasons for this discrepancy are unknown, we found it intriguing that the vascular cells from the macrocirulation, PAECs, AOECs, and PASMCs were all nearly 100% positive for SNA1. We also recognize that collection of cells with trypsin may alter the glycocalyx, however we chose this method for two reasons. First, these data compare to previous reports of lectin analysis of pulmonary endothelium, and further, to compare our MPs we required isolated cells rather than intact monolayers. Our data also clearly show no alterations with trypsin treatment. To our knowledge this type of direct comparison has not been performed and these data provides unique insight and a novel marker for the microvasculature. While the AOECs and PASMCs were also highly positive for GS1, our data support the concept that cells only positive for GS1, but not SNA, are most likely microvascular in origin and cells strongly SNA1 positive are residents of the macrovasculature.

Our goal was to determine whether lectin analysis would identify MPs from specific vascular beds. The only significant staining we could identify that was similar to the parent cell was SNA 1for PASMC-MPs and AOEC-MPs, however while they reached significance the staining was not 100% as that of the parent cells. We found that the MPs from the MVECs and PAECs were not significantly positive for any specific lectin. It is intriguing that the MPs released have a unique glycocalyx from the parent cell. MPs are reportedly derived directly from the parent cell membrane, however this lack of consistency in the glycocalyx may suggest differently. On the one hand, these data would support the concept of an immature glycocalyx derived from an intracellular source. Previous work has suggested that intracellular vesicles have a glycocalyx and possibly that this glycocalyx is unique from that of the plasma membrane [51, 52]. Alternatively, there is some evidence in the literature for flipping of extracellular membrane (i.e. PS exposure) that may provide an explanation for our observations. *While on the strength of the data presented we cannot conclude derivation from an intracellular source or extracellular presentation of the membrane*, *it is interesting to speculate*.

Damage to the glycocalyx is implicated in vascular permeability and cigarette smoke causes endothelial dysfunction, endothelial permeability, and apoptosis [42, 48, 49]. However, the effects of cigarette smoke on vascular cell glycocalyx have not previously been determined. We found that cigarette smoke had no effects on the glycocalyx of the PASMCs or the MVECs, however there were alterations in the PAEC and AOEC glycocalyxes. HP staining of both of the macrovascular endothelial cell type increased in response to CSE treatment. HP binding is an indicator of a poor prognosis in a number of cancers, suggesting that expression of terminal alpha N-acetylgalactosamine residues may be indicative of cellular damage or transformation, and may indicate a susceptibility to cigarette smoke injury of the macrocirculation.

We also observed no significant changes in the lectin profile of MPs from the CSE treated cells. Overall, while we found that MPs are easily analyzed by lectin staining, we did not find enough significant differences in the MP profiles to determine their vascular segment derivation. However, a more detailed lectin binding study, or costly mass spectrometry, may further reveal the subtle nuances that would resolve the exact cellular origins of the microparticles. Nonetheless, this descriptive study has revealed that MPs released into the extracellular milieu do not express the same glycocalyx as their cell of origin, however these data may provide useful information for further characterization of the circulating microparticle population. This characterization is vital to the potential use of MPs as biomarkers in health and disease.

## Supporting Information

S1 Fig Gating strategy and representative dot plot. A) 1 μm Fluoresbrite plain microsphere calibration beads in PBS were used to set MP inclusion gate (Events <1 μm). B) Unstained sample. Unlabeled microparticles (MPs) were used to set a gate based on FITC channel fluorescence (Events <1 μm FITC +). Note counting bead population in P2. C) SNA stained MPs. Percentage of the MP population that stained with the lectin is indicated by percent positive.
(TIF)

S2 Fig CSE induces caspase activity. MVECs treated with either 1% CSE, 3% CSE or staurosporine (1 μM) for 1 hour in serum free media were analyzed for caspase-3 activity using the FLICA caspase activity kit. 1% CSE, 3% CSE, and staurosporine all significantly induced caspase-3 activity (Control = 16.6 ± 4.2, Staurosporine = 37.33 ± 1.1, 1% CSE = 34.4 ± 2.2, and 3% CSE = 47.3 ± 0.92; n = 3 and p≤ 0.05).
(TIFF)
