# Supplementary figures and images for: Lectin-Based Characterization of Vascular Cell Microparticle Glycocalyx
Source: PLoS One. 2015 Aug 14;10(8):e0135533. doi: 10.1371/journal.pone.0135533 (PMC4537305; doi:10.1371/journal.pone.0135533)

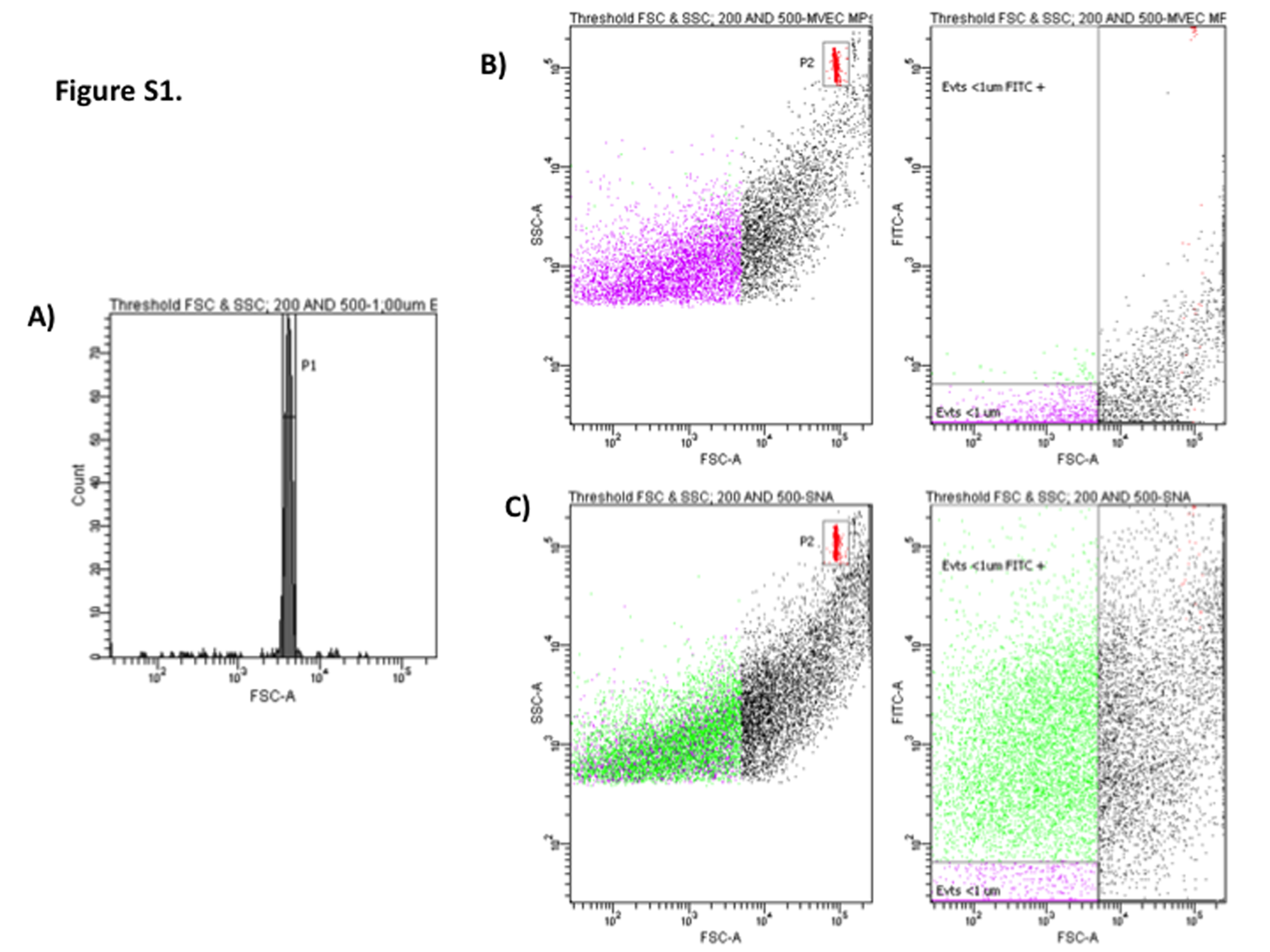

Supplement: S1 Fig — A) 1 μm Fluoresbrite plain microsphere calibration beads in PBS were used to set MP inclusion gate (Events <1 μm). B) Unstained sample. Unlabeled microparticles (MPs) were used to set a gate based on FITC channel fluorescence (Events <1 μm FITC +). Note counting bead population in P2. C) SNA stained MPs. Percentage of the MP population that stained with the lectin is indicated by percent positive. (TIF) [file pone.0135533.s001.tif]

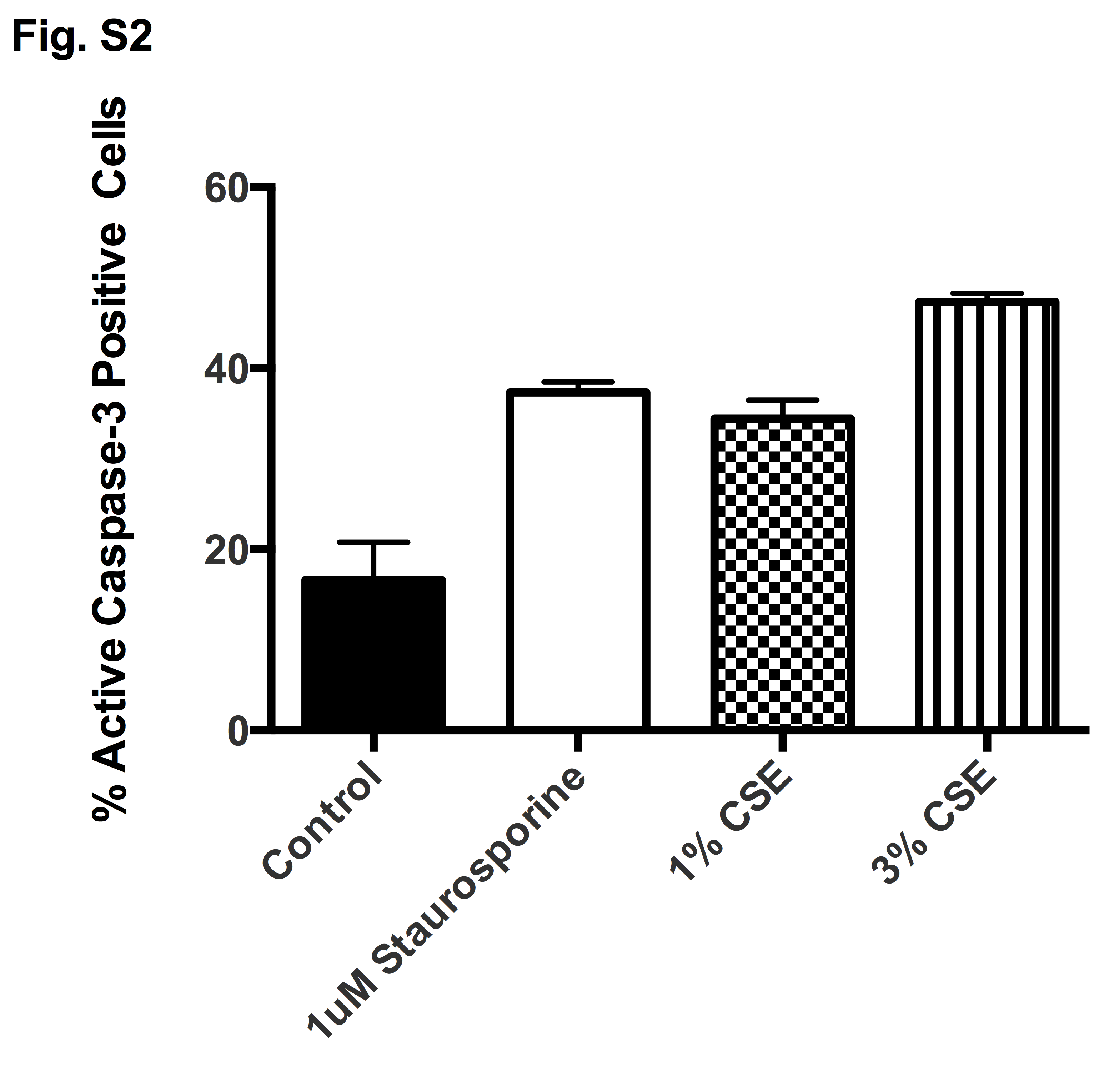

Supplement: S2 Fig — MVECs treated with either 1% CSE, 3% CSE or staurosporine (1 μM) for 1 hour in serum free media were analyzed for caspase-3 activity using the FLICA caspase activity kit. 1% CSE, 3% CSE, and staurosporine all significantly induced caspase-3 activity (Control = 16.6 ± 4.2, Staurosporine = 37.33 ± 1.1, 1% CSE = 34.4 ± 2.2, and 3% CSE = 47.3 ± 0.92; n = 3 and p≤ 0.05). (TIFF) [file pone.0135533.s002.tiff]
